# Culturomics revealed the bacterial constituents of the microbiota of a 10-year-old laboratory culture of planarian species *S. mediterranea*

**DOI:** 10.1038/s41598-021-03719-5

**Published:** 2021-12-21

**Authors:** Luis Johnson Kangale, Didier Raoult, Pierre-Edouard Fournier, Eric Ghigo

**Affiliations:** 1 Aix-Marseille Univ, IRD, AP-HM, SSA, VITROME, Marseille, France; 2 IHU-Méditerranée-Infection, Marseille, France; 3 Aix-Marseille Univ, IRD, AP-HM, MEPHI, Marseille, France; 4 Special Infectious Agents Unit, King Fahd Medical Research Center, King Abdulaziz University, Jeddah, Saudi Arabia; 5 TechnoJouvence, 19-21 Boulevard Jean Moulin, 13385 Marseille Cedex 05, France

**Keywords:** Microbial communities, Microbial ecology, Microbiome

## Abstract

The planarian species *Schmidtea mediterranea* is a flatworm living in freshwater that is used in the research laboratory as a model to study developmental and regeneration mechanisms, as well as antibacterial mechanisms. However, the cultivable microbial repertoire of the microbes comprising its microbiota remains unknown. Here, we characterized the bacterial constituents of a 10-year-old laboratory culture of planarian species *S. mediterranea* via culturomics analysis. We isolated 40 cultivable bacterial species, including 1 unidentifiable species. The predominant phylum is *Proteobacteria*, and the most common genus is *Pseudomonas*. We discovered that parts of the bacterial flora of the planarian *S. mediterranea* can be classified as fish pathogens and opportunistic human pathogens.

## Introduction

The microbiota is a complex ecology of an organism that varies greatly with time and lifestyles and between individuals, making it difficult to measure its implications in the organism’s immune response. Numerous model organisms (fruit fly, nematode, zebrafish, honey bee, hydra, squid)^1^ have been used to investigate the implications of the microbiota in the antimicrobial response. To date, there is increasing evidence that microbiomes play a role in animal immune function. Indeed, axenic animals show reduced expression of several immune effectors and are more susceptible to microbial pathogens than non-axenic animals. The efficiency of the immune response of the animal differs with the composition of the microbiota. Some microbial taxa or microbial communities promote inflammation, while others foster immune tolerance and host health^2,3^. Similarly, the diversity of the bacterial population of the microbiota causes the microbiota to play various roles in chronic inflammatory disease and autoimmune diseases^4,5^, in oncogenesis and tumour progression^6^, and in limiting colonization and invasion by microbes (bacteria, fungi, parasites) through the alteration of nutrient metabolite production or by secreting antimicrobial peptides^7,8^. In addition, microbiota flora participate in the production of various metabolites^9,10^, which are an important source of energy that improve the function of the intestinal barrier^11,12^.

Immortal planaria (Plathyhelminthes) are known for its ability to regenerate an entire organism from a tissue fragment. The regeneration ability of planarians has been intensively investigated^13–15^. Planarians have a worldwide distribution and live in a wide range of natural habitats, such as lakes, ponds, and rivers^16^. In the environment, planarians exist primarily as flesh-eating animals, though they also feed on detritus, fungi, and bacteria^17^. Due to their diet and habitat, planarians are exposed to a wide range of microbes and are able to survive this exposure. The ease of handling planarians and the application of loss-of-function genetic approaches in these organisms make them a valuable model system to investigate immune response^18^.

The use of the laboratory planarian species *Schmidtea mediterranea* has shown that planarians are powerful tools to identify evolutionarily conserved antibacterial response mechanisms. Indeed, the use of *Schmidtea mediterranea* has revealed that planarians represent a remarkable system with an unmatched capacity to fight infectious agents, including *Staphylococcus aureus, Mycobacterium tuberculosis, and Legionella pneumophila*, indicating the presence of remarkably efficient but uncharacterized innate immunity^18^. Notably, it has also been reported that planarians are able to overcome fungal infection^19^. Several new components of the innate immune system that are conserved in humans and absent from Ecdysozoa (e.g., flies and nematodes) were discovered by studying this model organism^18,20^. As an example, *Membrane Occupation and Recognition Nexus* (*MORN*) *repeat-containing-2* (*MORN2*) has been identified to be a component of LC3 (light chain 3)-associated phagocytosis^18^. Also the role of leukotriene A4 hydrolase, a pro-inflammatory lipid mediator^21^ timeless (circadian clock machinery)^22^ in *S. aureus* clearance has been demonstrated. *In-silico* analysis revealed that despite the identification and characterization of the repertoire of TIR domain-containing proteins in planarian species *S. mediterranea*, TLRs are absent from *S. mediterranea*^23^*,* and planarian stem cells are able to drive a sensitized antibacterial response through axis PGRP-2/setd8 methyltransferase signalling^24^. Very recently, it has been demonstrated that GILT (gamma interferon inducible lysosomal thiol reductase) is crucial to the antimicrobial response of planarians against gram-negative microbes^25^.

However, the use of planarians for studies of antimicrobial response requires a deeper knowledge of their microbial repertoire. Interestingly, all these studies have been performed using planarians kept in the laboratory for several years^18,19,22–24^. The contribution of the microbiota to the antimicrobial response of laboratory strains of planarians has not been investigated.

Microbiota can be investigated using metagenomics and/or culturomics. Metagenomics allows uncultured bacteria to be identified. It connects microbial signatures with a physiological condition or disease because the abundance of each taxon can be determined. Moreover, microbial species can be grouped without taxonomic assignment. Shotgun sequencing enables us to define the function of microbial communities via the analysis of genomes and coding capacity. Although metagenomics enables uncultured bacteria to be identified, in contrast to culturomics, metagenomics cannot allow functional analysis since bacteria are not cultivated. New species cannot be taxonomically validated since functional analysis cannot be performed, and bacterial strains cannot be officially deposited. Moreover, methods used for metagenomics are limited by the heterogeneity of the protocols used. As Lagier et al*.*^26^ wrote, “discrepant results can be obtained depending on the method used to extract DNA or the primers that are used for amplification. The variety of methodologies proposed for bioinformatics analyses (for example, operational taxonomic unit clustering, taxonomic assignment or statistical analysis) can substantially affect the results. Sequencing methods cannot discriminate between live bacteria and transient DNA, and despite recent progress, they cannot easily detect minority populations”.

In contrast to metagenomics, culturomics is a culture-based approach that uses multiple culture conditions, matrix-assisted laser desorption-ionization time of flight-mass spectrometry (MALDI-TOF MS) and complete 16S rRNA gene sequencing for the identification of bacterial species^26^. In addition, since bacteria are cultivated, culturomics allows for functional analysis, new species can be taxonomically validated, and new bacterial species can be officially deposited.

The goal of our study is to report the cultivable bacteria comprising the microbiota of a 10-year-old laboratory planarian reference species, *Schmidtea mediterranea,* to define a standard microbiota. This will, in the near future, give the scientific community the possibility to investigate, through the use of laboratory *Schmidtea mediterranea*, the implications of the cultivable bacteria detected in the antimicrobial response of planarians. For this purpose, the microbiota of laboratory *Schmidtea mediterranea* strains was analysed by a culturomic approach.

Our data highlight the presence of 40 bacterial species in the 10-year-old laboratory planarian reference species *Schmidtea* *mediterranea* used to investigate antimicrobial response. One species remains to be identified, and 4 species have been described to date only in planarians. The predominant phylum in the microbiota flora of *S. mediterranea* is *Proteobacteria,* and the most common genus is *Pseudomonas*. The flora of laboratory *Schmidtea mediterranea* strain is shared with several simple animal models, along with the environment, fish, and humans.

## Results

**Figure 1:**
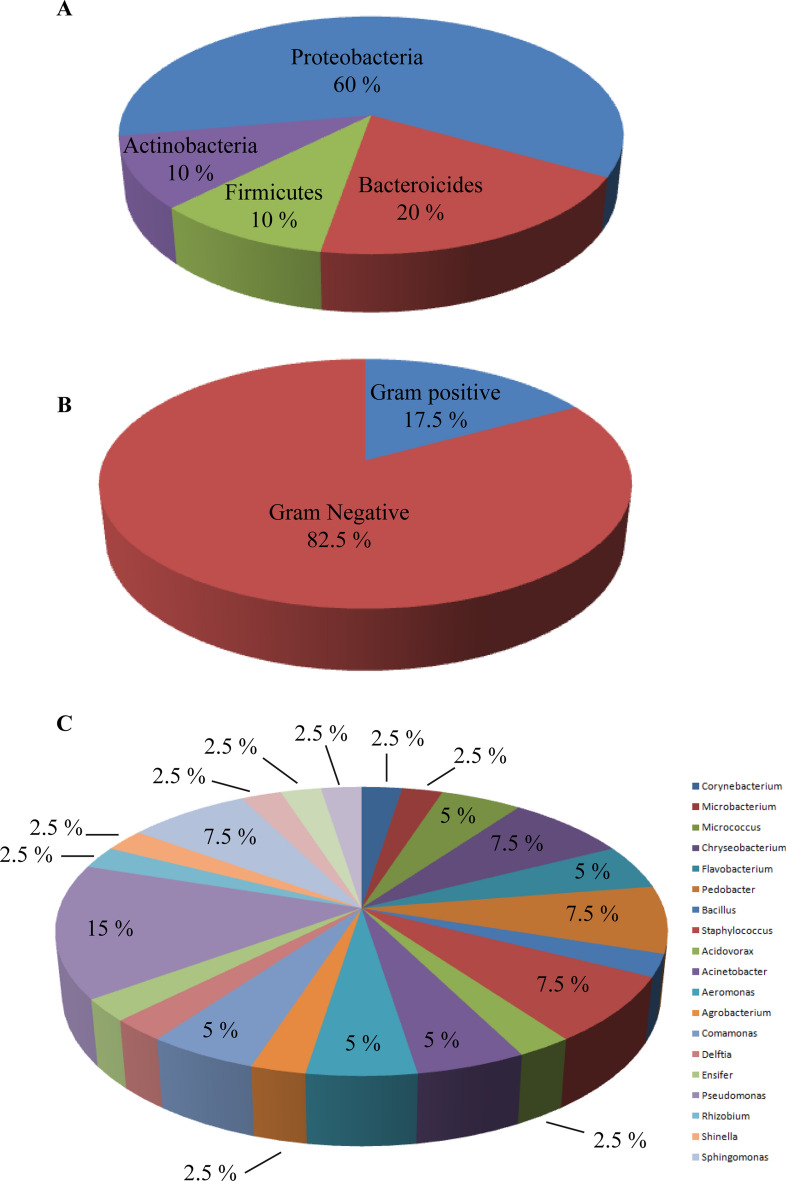
Bacterial composition of the microbiota of the laboratory strain *S. mediterranea* starved for 2 weeks. (**A**) Culturomics analysis of *S. mediterranea* followed by identification by MALDI TOF and 16S RNA sequencing revealed the phyla of the bacteria forming the microbiota of the animals (see also Table S1). (**B**) The nature of the bacterial membrane was determined by gram staining colouration. (**C**) The representativeness of each bacterial species is illustrated. Five experiments were each performed on ten individual worms.

Using the culturomics approach to determine the bacterial communities of *S. mediterranea* starved for 2 weeks allowed us to isolate and identify (by MALDI-TOF or 16 s RNA sequencing) a total of 40 bacterial species after 1 to 4 days of culturing at temperatures of 19 °C, 28 °C, and 37 °C on the following media: LB agar, COS, and BCYE. These bacterial species corresponded to 4 bacterial phyla: *Proteobacteria*, *Bacteroidetes*, *Actinobacteria*, and *Firmicutes* (Fig. 1A, Table 1, Table S1). The phenotypic characteristics of the colonies of the identified bacterial species are reported in Table S2 along with their growth conditions. In the laboratory *S. mediterranea*, the dominant phylum, accounting for 60% of all phyla, is *Proteobacteria*, and the less represented phyla (10%) are *Actinobacteria* and *Firmicutes*. The phylum *Bacteroides* accounted for 20% (Fig. 1A, Table 1). There was a predominance of gram-negative bacteria (82.5%), while gram-positive bacteria represented 17.5% of the bacterial population (Fig. 1B). The most common genus found in the bacterial population was *Pseudomonas*, accounting for 15% (Fig. 1C, Table 1).

**Table 1:** Bacterial composition of the microbiota of the laboratory strain *S. mediterranea* starved for 2 weeks.

*Species*	Phyla	Gram
*Corynebacterium lipophiloflavum*	Actinobacteria	+
*Microbacterium oxydans*	Actinobacteria	+
*Micrococcus luteus*	Actinobacteria	+
*Micrococcus yunnanensis*	Actinobacteria	+
*Chryseobacterium balustinum*	Bacteroidetes	−
*Chryseobacterium schmidteae*	Bacteroidetes	−
*Chryseobacterium scophthalmum*	Bacteroidetes	−
*Flavobacterium oncorhynchi*	Bacteroidetes	−
*Flavobacterium tructae*	Bacteroidetes	−
*Pedobacter ghigonii*	Bacteroidetes	−
*Pedobacter schmidteae*	Bacteroidetes	−
*Pedobacter wanjuense*	Bacteroidetes	−
*Metabacillus schmidteae*	Firmicutes	−
*Staphylococcus capitis*	Firmicutes	+
*Staphylococcus epidermidis*	Firmicutes	+
*Staphylococcus haemolyticus*	Firmicutes	+
*Acidovorax wautersii*	Proteobacteria	−
*Acinetobacter bereziniae*	Proteobacteria	−
*Acinetobacter guillouiae*	Proteobacteria	−
*Aeromonas hydrophila*	Proteobacteria	−
*Aeromonas veronii*	Proteobacteria	−
*Agrobacterium tumefaciens*	Proteobacteria	−
*Comamonas aquatilis*	Proteobacteria	−
*Comamonas testosteroni*	Proteobacteria	−
*Delftia acidovorans*	Proteobacteria	−
*Ensifer adhaerens*	Proteobacteria	−
*Pseudomonas anguilliseptica*	Proteobacteria	−
*Pseudomonas brenneri*	Proteobacteria	−
*Pseudomonas fluorescens*	Proteobacteria	−
*Pseudomonas gessardii*	Proteobacteria	−
*Pseudomonas huaxiensis*	Proteobacteria	−
*Pseudomonas* sp. Marseille-Q1929	Proteobacteria	−
*Rhizobium giardinii*	Proteobacteria	−
*Shinella zoogloeoides*	Proteobacteria	−
*Sphingomonas bisphenolicum*	Proteobacteria	−
*Sphingomonas ginsenosidimutans*	Proteobacteria	−
*Sphingomonas paucimobilis*	Proteobacteria	−
*Variovorax paradoxus*	Proteobacteria	−
*Vogesella urethralis*	Proteobacteria	−
*Herminiimonas contaminans*	Proteobacteria	−

This table lists the bacteria identified in the planarian *S. mediterranea* microbiota as follows: species, phyla, and nature of the membrane analysed by gram staining.

**Figure 2:**
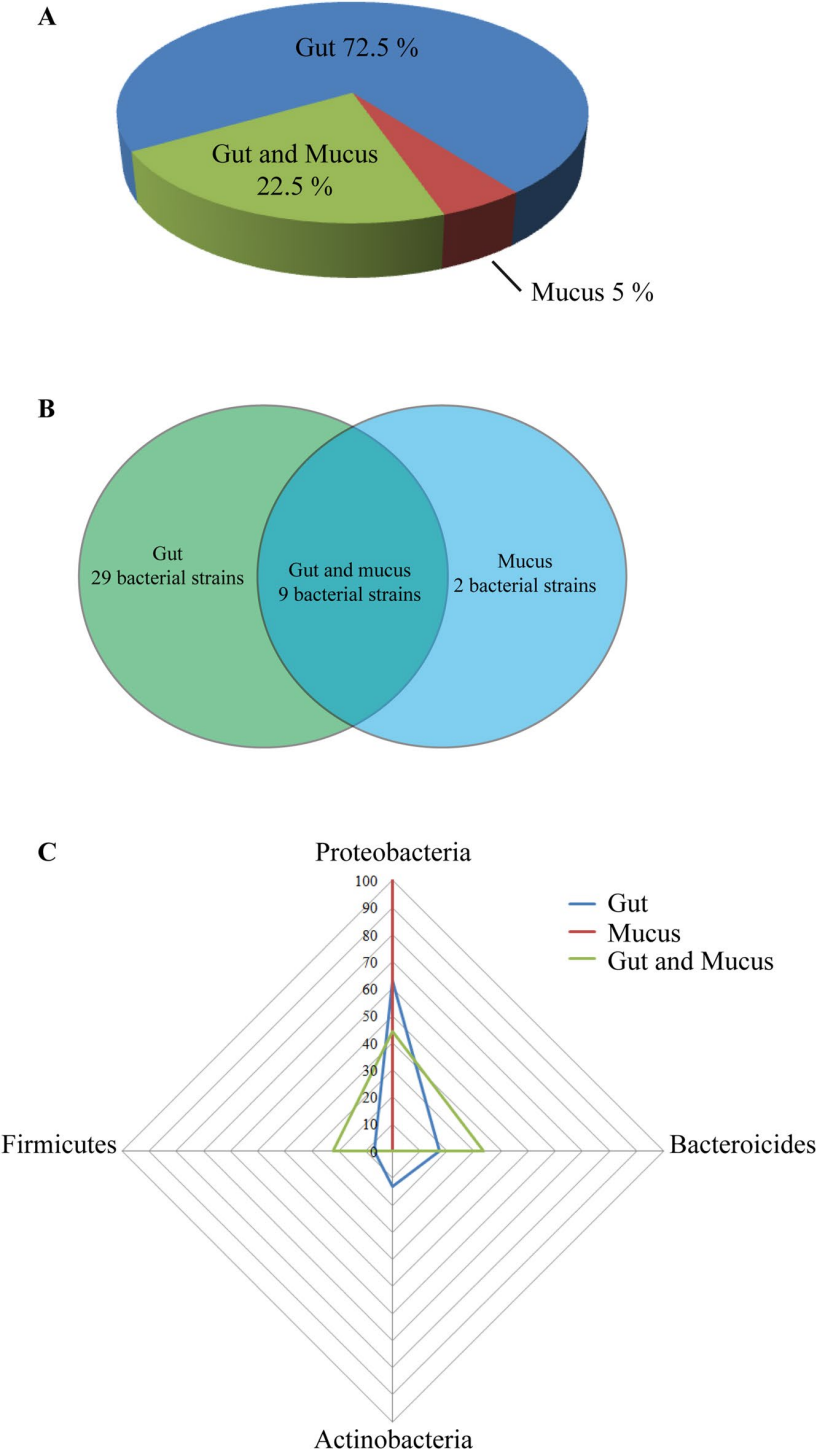
Bacterial distribution of the laboratory strain *S. mediterranea* starved for 2 weeks. (**A**) Distribution of the bacterial communities of the planarian *S. mediterranea*, (**B**) Venn diagram highlighting the bacterial number present in the gut and mucus epidermal (noted here as mucus), and the gut and epidermal mucus combined of *S. mediterranea*, (**C**) representation of the distribution of the bacterial phyla in the gut, epidermal mucus (noted here as mucus), and the gut and epidermal mucus combined of *S. mediterranea*. Five experiments were each performed on ten individual worms (see also Table S3). Identical results were obtained for each experiment and each worm tested.

The bacterial communities of the planarian *S. mediterranea* were distributed as follows: 72.5% of bacterial species colonized the gut, 5% the mucus, and 22.5% both the mucus and gut (Fig. 2A, Table S3). Two gram-negative bacterial species, *Vogesella urethralis* and *Shinella zoogloeoides*, both of which are from the phylum *Proteobacteria*, are specific to the *S. mediterranea* mucus (Fig. 2B,C, Table S3). Whereas four phyla (29 bacterial strains in total) were found within the gut of *S. mediterranea*, only three phyla (*Proteobacteria*, *Bacteroidetes*, and *Firmicutes*, represented by 9 bacterial strains) were present in both the gut and mucus (Fig. 2B,C, Table S3). Among the 40 bacterial species isolated, 1 is a nonclassified or putative new species of *Pseudomonas* from the phylum *Proteobacteria* and must be characterized by taxonogenomic methods in the near future. In addition, 4 bacterial species have been recently identified as new bacterial species: *Chryseobacterium schmidteae*^27^, *Pedobacter ghigonii*^28^, and *Pedobacter schmidteae*^29^ from the phylum *Bacteroidetes,* as well as *Metabacillus schmidteae* from the phylum *Firmicutes*^30^.

**Figure 3:**
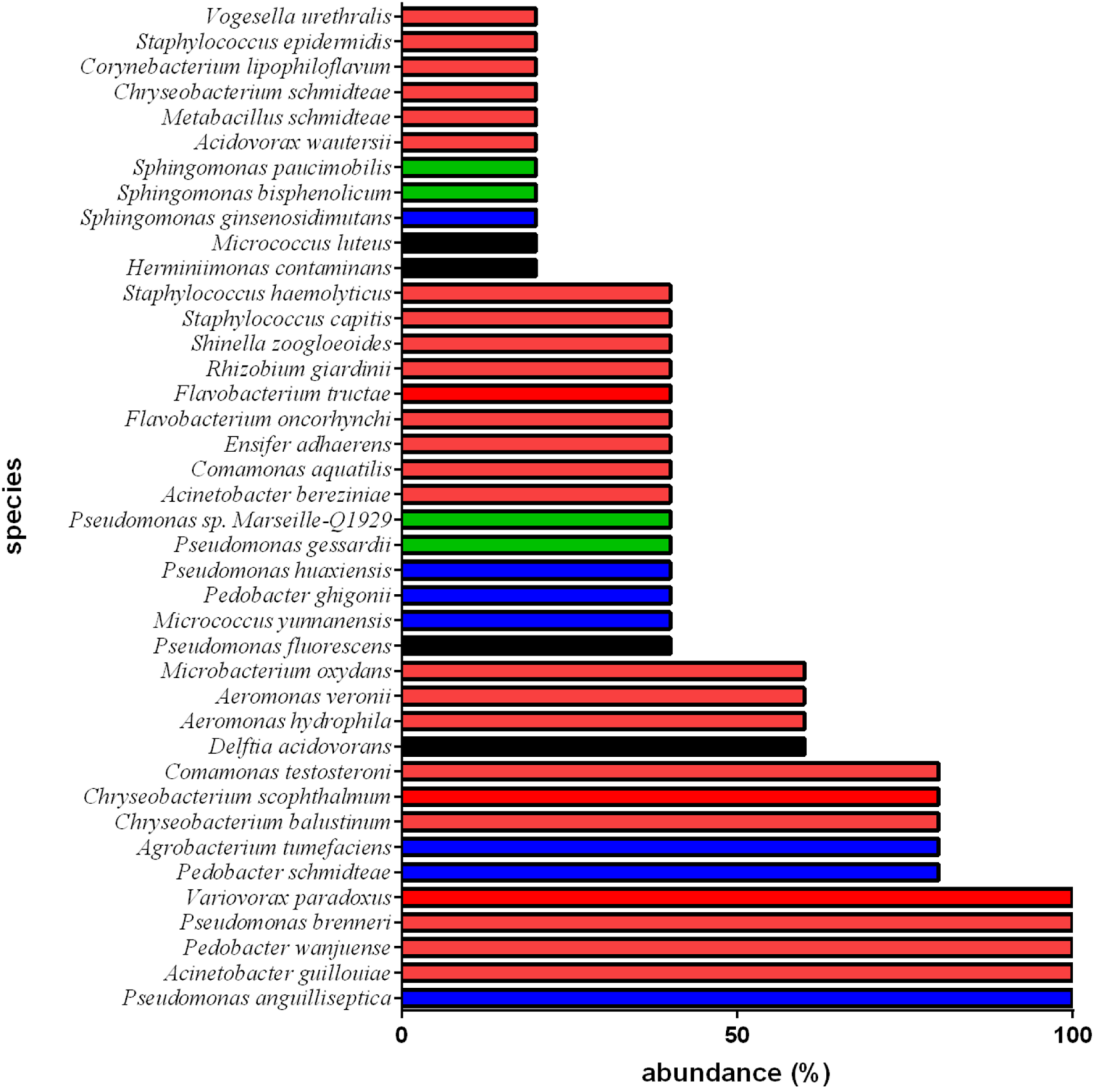
Abundance of the bacterial species identified in the laboratory strain of *S. mediterranea* starved for 2 weeks. The abundance is expressed as a percentage of the bacterial species. Phyla are shown as follows: Proteobacteria in red, Firmicutes in green, Bacteroidetes in blue, and Actinobacteria in black. Five experiments were each performed on ten individual worms (see also Table S4). Identical results were obtained for each experiment and each worm tested.

Next, we analysed the abundance of the identified bacterial species (Fig. 3, Table S4). Fifteen species were detected in 40% of the *S. mediterranea* planarians tested, and 11 were detected in 20% of *S. mediterranea* planarians. The other species are detected in between 60 and 100% of the planarians. This abundance was not correlated with the phylum or the localization of the bacteria within planarians (Fig. 3).

Next, we compared the microbiota of *S. mediterranea* starved for 1 week, 1 weeks, and four weeks (Table 2). After 1 week of starvation, we identified 14 bacterial strains. This is in contrast to 2 and 4 weeks of starvation, after which we found 40 bacterial strains. Bacterial strains detected after 1 week of starvation were also detected after 2 and 4 weeks of starvation. The bacterial strains identified after 2 and 4 weeks of starvation were the same. After 1 week of starvation, the phyla *Proteobacteria* and *Bacteroidetes* represented 50 and 43%, respectively, of the bacterial strains present, while *Actinobacteria* represented less than 10%. After 1 week of starvation, the *Firmicutes* are not represented like they are after 2 weeks and 4 weeks of starvation.

**Table 2:** Bacterial composition of the microbiota of the laboratory strain *S. mediterranea* starved for different duration.

1 week of starvation	2 weeks of starvation	4 weeks of starvation
*Acinetobacter guillouiae*	*Acidovorax wautersii*	*Acidovorax wautersii*
	*Acinetobacter bereziniae*	*Acinetobacter bereziniae*
	*Acinetobacter guillouiae*	*Acinetobacter guillouiae*
	*Aeromonas hydrophila*	*Aeromonas hydrophila*
	*Aeromonas veronii*	*Aeromonas veronii*
*Agrobacterium tumefaciens*	*Agrobacterium tumefaciens*	*Agrobacterium tumefaciens*
*Chryseobacterium balustinum*	*Chryseobacterium balustinum*	*Chryseobacterium balustinum*
	*Chryseobacterium schmidteae*	*Chryseobacterium schmidteae*
*Chryseobacterium scophthalmum*	*Chryseobacterium scophthalmum*	*Chryseobacterium scophthalmum*
	*Comamonas aquatilis*	*Comamonas aquatilis*
*Comamonas testosteroni*	*Comamonas testosteroni*	*Comamonas testosteroni*
*Corynebacterium lipophiloflavum*	*Corynebacterium lipophiloflavum*	*Corynebacterium lipophiloflavum*
*Delftia acidovorans*	*Delftia acidovorans*	*Delftia acidovorans*
	*Ensifer adhaerens*	*Ensifer adhaerens*
*Flavobacterium oncorhynchi*	*Flavobacterium oncorhynchi*	*Flavobacterium oncorhynchi*
*Flavobacterium tructae*	*Flavobacterium tructae*	*Flavobacterium tructae*
	*Herminiimonas contaminans*	*Herminiimonas contaminans*
	*Metabacillus schmidteae*	*Metabacillus schmidteae*
	*Microbacterium oxydans*	*Microbacterium oxydans*
	*Micrococcus luteus*	*Micrococcus luteus*
	*Micrococcus yunnanensis*	*Micrococcus yunnanensis*
*Pedobacter ghigonii*	*Pedobacter ghigonii*	*Pedobacter ghigonii*
	*Pedobacter schmidteae*	*Pedobacter schmidteae*
*Pedobacter wanjuense*	*Pedobacter wanjuense*	*Pedobacter wanjuense*
*Pseudomonas anguilliseptica*	*Pseudomonas anguilliseptica*	*Pseudomonas anguilliseptica*
*Pseudomonas brenneri*	*Pseudomonas brenneri*	*Pseudomonas brenneri*
	*Pseudomonas fluorescens*	*Pseudomonas fluorescens*
	*Pseudomonas gessardii*	*Pseudomonas gessardii*
	*Pseudomonas huaxiensis*	*Pseudomonas huaxiensis*
	*Pseudomonas sp. Marseille-Q1929*	*Pseudomonas* sp. *Marseille-Q1929*
	*Rhizobium giardinii*	*Rhizobium giardinii*
	*Shinella zoogloeoides*	*Shinella zoogloeoides*
	*Sphingomonas bisphenolicum*	*Sphingomonas bisphenolicum*
	*Sphingomonas ginsenosidimutans*	*Sphingomonas ginsenosidimutans*
	*Sphingomonas paucimobilis*	*Sphingomonas paucimobilis*
	*Staphylococcus capitis*	*Staphylococcus capitis*
	*Staphylococcus epidermidis*	*Staphylococcus epidermidis*
	*Staphylococcus haemolyticus*	*Staphylococcus haemolyticus*
*Variovorax paradoxus*	*Variovorax paradoxus*	*Variovorax paradoxus*
	*Vogesella urethralis*	*Vogesella urethralis*

This table lists the bacteria identified in the planarian *S. mediterranea* microbiota starved for 1 week, 2 weeks, and 4 weeks as follows: species, phyla, and nature of the membrane analysed by gram staining. The distribution remained unchanged after 2 weeks of starvation. Five experiments were each performed on ten individual worms (see also Table S3). Identical results were obtained for each experiment and each worm tested.

**Figure 4:**
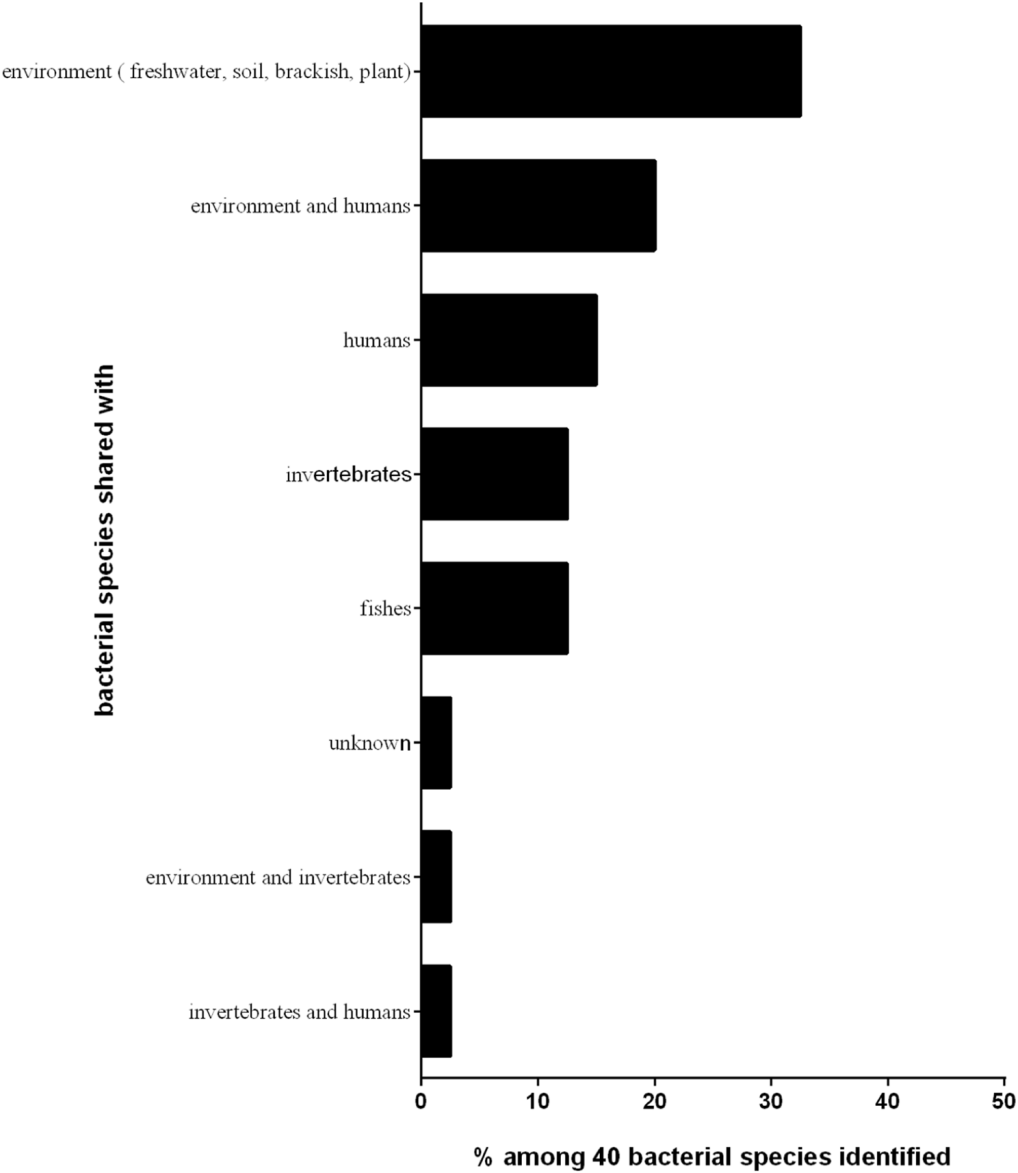
The bacterial species identified in the laboratory strain *S. mediterranea* starved for 2 weeks are shared with the environment, vertebrates, and invertebrates. Bibliographic analysis allowed us to illustrate the potential sharing of the bacterial strains forming *S. mediterranea* microbiota (see also Table 3).

Finally, we analysed whether the microorganisms identified in the laboratory *S. mediterranea* strain were shared with the environment, invertebrates, fish, and humans (Fig. 4, Table 3). We found that 32.50% of the bacteria are shared with the environment (freshwater, soil, brackish water, and plants), 20% are shared with the environment and humans, 15% are shared with humans^31–45^, 12.5% are shared with fish and have also been described as fish pathogens^31–34^, 12.50% are shared with invertebrates^27–30,46–48^, and 2.5% (*Herminiimonas* sp. *Marseille-P9896*) cannot be categorized because they are uncharacterized bacterial species. This last point illustrates the complexity of the microbiota of the laboratory *S. mediterranea* strain.

**Table 3:** The bacterial species identified in the laboratory strain *S. mediterranea* starved for 2 weeks are shared with environment, vertebrates and invertebrates.

Species	Share with	References
*Acidovorax wautersii*	Environment and human	^36^
*Acinetobacter guillouiae*	Environment and human	^80^
*Aeromonas hydrophila*	Environment and human	^81^
*Comamonas testosteroni*	Environment and human	^82,83^
*Delftia acidovorans*	Environment and human	^84,85^
*Micrococcus luteus*	Environment and human	^86,87^
*Sphingomonas paucimobilis*	Environment and human	^88,89^
*Staphylococcus haemolyticus*	Environment and human	^90^
*Pseudomonas brenneri*	Environment	^91,92^
*Variovorax paradoxus*	Environment	^93^
*Pseudomonas gessardii*	Environment	^94^
*Pseudomonas fluorescens*	Environment	^95^
*Pseudomonas huaxiensis*	Environment	^96^
*Pedobacter wanjuense*	Environment	^97^
*Shinella zoogloeoides*	Environment	^98^
*Sphingomonas bisphenolicum*	Environment	^99^
*Agrobacterium tumefaciens*	Environment	^100^
*Ensifer adhaerens*	Environment	^101^
*Rhizobium giardinii*	Environment	^102^
*Micrococcus yunnanensis*	Environment	^103^
*Sphingomonas ginsenosidimutans*	Environment	^104^
*Pseudomonas anguilliseptica*	Fish	^105^
*Flavobacterium oncorhynchi*	Fish	^32^
*Flavobacterium tructae*	Fish	^31^
*Chryseobacterium balustinum*	Fish	^106^
*Chryseobacterium scophthalmum*	Fish	^106^
*Vogesella urethralis*	Human	^107^
*Microbacterium oxydans*	Human	^108^
*Corynebacterium lipophiloflavum*	Human	^109^
*Staphylococcus capitis*	Human	^110^
*Staphylococcus epidermidis*	Human	^111^
*Acinetobacter bereziniae*	Human	^112^
*Aeromonas veronii*	Invertebrate and human	^48^
*Pedobacter schmidteae*	Invertebrate	^29^
*Pedobacter ghigonii*	Invertebrate	^28^
*Chryseobacterium schmidteae*	Invertebrate	^27^
*Metabacillus schmidteae*	Invertebrate	^30^
*Herminiimonas contaminans*	Invertebrates	^47^
*Comamonas aquatilis*	Environment and invertebrate	^46,49^
*Pseudomonas* sp. *Marseille-Q1929*	Unknown	

Bibliographic analysis allowed us to illustrate the potential sharing of the bacterial strains forming *S. mediterranea* microbiota.

Taken together, these data show that the predominant phylum in the *S. mediterranea* laboratory planarian flora is *Proteobacteria*, that the most common genus is *Pseudomonas*, and that planarians share microorganisms with both invertebrates and vertebrates.

## Discussion

Analysis of the microbiome of the laboratory strain planarian species *S. mediterranea* using culturomics methods allowed us to identify 40 cultivable bacterial species forming the microbiota repertoire of *S. mediterranea*, most of which are found in the planarian gut. Among the 40 isolated bacterial species, we identified one non classified bacterial species (*Pseudomonas* sp.) that needs to be taxonomically and biochemically characterized in further work, four bacterial species (*Pedobacter schmidteae*, *Pedobacter ghigonii*, *Chryseobacterium schmidteae*, *Metabacillus schmidteae*) that to date have been identified only in planarians^27–30^, and one bacterial species (*Comamonas aquatilis*) that has been identified in both pond water and *S. mediterranea*^46,49^. In the calf liver used to feed *S. mediterranea*, we detected several bacterial species, including *Brochothrix thermosphacta*, *Lactococcus piscium*, *Pseudomonas frederiksbergensis*, *Pseudomonas gessardii*, *Serratia proteamaculans*, *Staphylococcus hominis*, and *Pseudomonas azotoformans* (Table S5). None of these were found in the planarian *S. mediterranea*, suggesting that they were thus eliminated by planarians. The division of bacterial species between the planarian gut and epidermal mucus remains difficult to interpret. We cannot be sure of 100% bacterial species repartition because bacteria can be regurgitated by planarians through the pharynx. This phenomenon most likely occurs for the following bacteria, which are primarily from the phylum *Proteobacteria*: *Aeromonas veronii*, *Chryseobacterium scophthalmum*, and *Pseudomonas brennerii*, along with *Pseudomonas anguilliseptica* bacteroidetes, because this bacterial strain are detected in water containing planarians (Table S6). Notably, no bacteria were detected in the water control, which does not contain planarians.

We also observed a variation in the microbiota composition as a function of starvation time. Indeed, whereas only 14 bacteria were detected after 1 week of starvation, 40 bacteria were detected after 2 and 4 weeks of starvation. Interestingly, the bacterial composition remained the same between 2 and 4 weeks of starvation, and the bacteria detected after one week of starvation were also found in the microbiota after 2 and 4 weeks of starvation. The bacterial distribution remained unchanged after 2 weeks of starvation. This microbiota evolution can be explained in several ways. First, after 1 week of starvation, the number of bacteria present in the worms was too low to allow for the detection of all the bacterial strains. Second, the bacteria present in the liver, although eliminated by planarians, can shape the composition of the microbiota after feeding by promoting the growth of different bacterial strains. Third, the 14 bacteria detected in planarians after 1 week of starvation promoted the growth of the other strains. Fourth, feeding induces the growth of planarians, thus modulating tissue homeostasis and regeneration; it cannot be ignored that feeding allows the development of several bacterial strains from planarian microbiota.

*Firmicutes* and *Actinobacteria* represent only 10% of the bacterial population found in the planarian *S. mediterranea*; *Proteobacteria* (60%) and *Bacteroidetes* (20%) are the predominant phyla in the microbiota. *Proteobacteria* is one of the largest bacterial phyla, with six classes and more than 116 families having been recognized (http://www.bacterio.net/). *Proteobacteria* are gram-negative bacteria and play numerous roles in diverse microbial ecosystems (aquatic, soil, plant, animal). *Proteobacteria* are involved in maintaining homeostasis of the gastrointestinal tract anaerobic environment and, thus, in the stability of the strictly anaerobic microbiota. Members of the phylum *Bacteroidetes* are known to be involved in the synthesis of short-chain fatty acids such as butyrate, propionate, and acetate, which are rich sources of energy for the host^50,51^. They also participate in carbohydrate metabolism by expressing enzymes such as glycosyl transferases, glycoside hydrolases, and polysaccharide lyases. Interestingly, *Bacteroidetes* have been shown to synthesize conjugated linoleic acid, which is reported to have immunomodulatory properties^52–54^. In addition, the bacteria present in the planarian microbiome are mostly gram-negative, and gram-negative organisms produce antimicrobial peptides^55^. Thus, it can be hypothesized that *Bacteroidetes* and other gram-negative bacteria, such as *Proteobacteria* bacteria, participate strongly in the control of the antimicrobial properties of planarians via antimicrobial peptides and immune regulation.

It is difficult to consider and discuss each bacterial strain found in *S. mediterranea* and to compare them with the microbiota of other simple animal models or humans. However, it has been reported that the *Proteobacteria* is the dominant microbial phylum found in *Danio rerio*, *Apis melifera*, and *Caenorhabditis elegans*^1,56^. The predominance of *Proteobacteria* has also been reported in the microbiota of another planarian species called *Dugesia japonica*^57^, as well as in the fruit fly, *Drosophila. melanogaster*^58^, and *Hydra oligactis*^59^. Notably, *Firmicutes* are also the predominant phyla for *C. elegans*. The other phyla, *Bacteroidetes* and *Actinobacteria*, were not found in the simple animal models cited above. *Stenostomum leucops*, belonging to Catenulida within the phylum Platyhelminthes, are tiny planarians of the phylum *Platyhelminthes* that reproduce asexually and have a lifestyle close to *S. mediterranea*. They also share several microorganisms with *S. mediterranea*, including *S. epidermidis*, *A. tumefaciens*, *E. adhaerens*, *P. fluorescens*, and *V. paradoxas*^60^*.*

In contrast to that of planarian species *S. mediterranea*, the normal human gut microbiota is predominantly composed of two major phyla, *Bacteroidetes* and *Firmicutes*, followed by *Actinobacteria* and *Verrucomicrobia*. *Verrucomicrobia* were not detected in *S. mediterranea*. In humans, the gut microbiota contains only a minor proportion of the phylum *Proteobacteria*. It has been shown that an increase in the *Proteobacteria* phylum is a potential signature of dysbiosis and indicates a higher risk of disease^61^. In humans, the presence of *Proteobacteria* within the microbiota is associated with an adaptation of the gut microbial community to the host’s diet, which could improve the ability of the host to harvest energy from indigestible polysaccharides^61^. Accordingly, we can hypothesize that the presence of large amounts of *Proteobacteria* in *S. mediterranea* might be associated with the capacity of planarians to adapt their body size to food availability. Indeed, in the absence of food, planarian size decreases, whereas in the presence of sufficient amounts of food, their body size increases greatly^62,63^.

In the planarian *S. mediterranea*, the major genus found is *Pseudomonas*, unlike in *Drosophila*, where the most common genus is *Klebsiella*^64^. Several bacterial species found in planarians are common to the *Drosophila* microbiota, such as *Micrococcus luteu*s, *Micrococcus yuennansis*, and *Microbacterium oxydans*. In *S. mediterranea*, *Aeromonas veronii* was also detected*.* Although its function remains unknown, it has been shown that *Aeromonas veronii* plays a crucial role in the immune response of *D. rerio* for upregulating neutrophil abundance, which leads to a downmodulation of inflammation^1^.

In 2016, Arnold et al.^65^ reported that planarian *S. mediterranea* microbiota analysed by metagenomics contain more than 300 bacterial strains. Here, in our study, we reported 40 bacterial strains, among which we identified and cultivated 14 bacterial strains described by Arnold et al., including *M. luteus*, *M. yunnanensis*, *S. capitis*, *S. epidermitis*, *A. guillouiae*, *A. tumefaciens*, *C. testosteroni*, *D. acidovorans*, *P. anguilliseptica*, *P. brenneri*, *P. fluorescens*, *P. gessardii*, *S. ginsenosidimutans*, and *V. paradoxus*. Such a disparity can be explained by the methodology used; metagenomics highlights any nucleotide sequence related to a bacterial strain, but the cultivability of the strain remains unknown. In addition, as shown by Arnold et al., the methods of culturing *S. mediterranea* can easily change the microbiota composition^65^. The role of the feeding (beef liver vs. calf liver) cannot be excluded. Thus, the composition of microbiota from the same species of planarians, here *S. mediterranea* CI4W, but kept in another laboratory will be affected by the methodology of bacterial detection and the culture conditions. A similar situation has been described for the microbiota of *D. melanogaster*^66^.

We have also observed that the bacteria comprising the microbiota of *S. mediterranea* are shared with the environment (soil, water, brackish water, sewer, and plants) as well as with fish, which is in accordance with the lifestyle of planarians. Indeed, planarians are zoophages and live in water. We also found bacteria that are shared with humans. Indeed, 16 bacterial strains have been described as opportunistic human pathogens, which represent 40% of the *S. mediterranea* flora and are responsible for diarrhoea, bacteremia, and endocarditis. Some of them have been reported to cause nosocomial infections, such as *Acinetobacter guillouiae*, or infection in immunocompromised people, such as *Micrococcus luteus*, which is involved in bacteraemia associated with intravascular catheters and endocarditis, peritonitis, ocular infections, and urinary tract infections. We also identified *Aeromonas veronii*, which is commonly hosted by leeches and is known to be responsible for gastroenteritis in humans^67^. The virulence and opportunistic capacity remain unclear for some of the bacterial species identified, such as *Pseudomonas fluorescens* and *Comamonas testosterone*, which might be a cause of bacteraemia or gastroenteritis^68,69^. As in *D. melanogaster*, we detected bacteria that have been described to be opportunistic human pathogens, such as *Micrococcus luteus*. This is also an important pathogen for aquatic animals^70^, but it is also a probiotic which promotes the growth of Nile tilapia *Oreochromis niloticus*”^71^. We also found several *Staphylococcus* species responsible for bacteraemia, sepsis, and nosocomial infection. The planarian *S. mediterranea* microbiota flora also includes *Aeromonas veronii*, which is commonly hosted by leeches. Cases of infection, such as gastroenteritis, have been reported in people using leech therapy procedures^35^ or eating contaminated fishes^72^. Similarly, *Mycobacterium marinum* infections have been reported to be associated with the exposure of damaged skin to polluted water from fish pools or objects contaminated with infected fish^73^. Planarians are known to be fish tank invaders. Thus, although the planarian *S. mediterranea* is a free-living flatworm, it cannot be ignored that planarians are a reservoir or host of several human microbial pathogens that might be transmitted to predators of planarians or released in fish tanks contaminated with planarians.

Several studies suggest the role of probiotics in tissue homeostasis, as well as in tissue regeneration^74^, and that manipulation of the microbiome could be a way to resolve some tissue homeostasis deficiencies^75^. It has been shown that antibiotic treatment affects the planarian microbiota, which leads to an alteration of the regeneration process of *S. mediterranea*^65^. In *Dugesia japonica*, metabolites such as indole produced by the bacteria *Aquitalea* sp*.* delay the regeneration of the tissue after amputation^57^. Although there are contradictory and controversial findings, it appears that commensals, symbionts, and pathogens from the human cutaneous microbiome can play an important role in the resolution of nonhealing wounds^75^.

The role of the microbiota in the antimicrobial capacity of *S. mediterranea* and in their ability to have trained immunity remain to be elucidated. For this purpose, it is important to have information concerning the composition of the microbiota of the laboratory strain used for the experiments and to consider that a divergence in results can be caused by the mode of culture.

## Materials and methods

### Culture of the planarian species *Schmidtea mediterranea*

The *S. mediterranea* asexual clonal line ClW4^24^ was maintained at 18 °C in water. The water was first filtered through charcoal and ceramics with pores of 0.2 µm (manufactured by Fairey Industrial Ceramics Limited) and through a membrane of 0.2 µm (Thermo Scientific Nalgene Filtration Products) for 10 years. Microbiological analysis of the filtered water was performed by inoculation of 5% sheep blood-enriched Columbia agar plates (bioMérieux, Marcy l’étoile, France) with 25, 50, or 100 µL of filtered water. Inoculated plates were then incubated at 19, 28, or 37 °C for 4 days. Any bacteria were detected*.*

*S. mediterranea* were fed once per week with homogenized calf liver (batch 20118-5814, origin: Saprimex (a local supermarket), 13310 St Martin de Crau) and then starved 2 weeks prior to experiments. In some experiments, worms were starved for 1 or 4 weeks. The bacterial constituents of homogenized calf liver were defined by inoculation of 5% sheep blood-enriched Columbia agar plates (bioMérieux, Marcy l’étoile, France) with 25, 50, or 100 µL of homogenized calf liver. Inoculated plates were then incubated at 19, 28, or 37 °C for 1, 2, 3, and 4 days.

### Culturomics

Two-week starved planarians 0.4–0.6 mm in length were used for the experiments. Selected worms were placed on agar plates (13%) and pressed slightly to collect their epidermal mucus (also denoted as mucus in the manuscript). The recovered epidermal mucus (one planarian per sample) was mixed with sterile phosphate-buffered saline (PBS), and then 100 µL of sample was inoculated on 5% sheep blood-enriched Columbia agar (bioMérieux, Marcy l’étoile, France), buffered charcoal yeast extract (BCYE) (Oxoid Deutschland GmbH, Wesel, Germany), and lysogeny broth (LB) under anaerobic and aerobic conditions and incubated at 19, 28, or 37 °C for 1, 2, 3, and 4 days^76^. The whole microbiota (gut and mucus) was then characterized by grinding one two-week-starved animal in PBS (one worm per sample). Homogenates were inoculated on 5% sheep blood-enriched Columbia agar (bioMérieux, Marcy l’étoile, France), BCYE (Oxoid Deutschland GmbH, Wesel, Germany), and lysogeny broth (LB) under anaerobic and aerobic conditions and then incubated at 19, 28 or 37 °C for 1, 2, 3, and 4 days.

### MALDI-TOF MS and bacterial identification

Individual bacterial colonies were collected every day for 4 days, and then each colony was identified by matrix-assisted laser desorption-ionization time-of-flight mass spectrometry (MALDI-TOF MS) (Microflex Spectrometer; Bruker Daltonics, Bremen, Germany) as previously described^77^. The obtained MALDI-TOF MS spectra were imported into MALDI Biotyper 3.0 software (Bruker Daltonics) and analysed against the reference bacterial spectral database. The MALDI Biotyper RTC software interprets the results according to predefined values, i.e., values between 2.00 ≤ species identified ≤ 3.00; of 1.70 ≤ probably identified ≤ 1.99 and 0.00 ≤ no identification ≤ 1.69. The unidentified colonies (with values from 0.00 to 1.99) were sequenced using the complete 16S rRNA gene.

### Sequencing of the 16S rRNA gene and bacterial identification

The unidentified bacterial colonies were cultured under the appropriate conditions, and the genomic DNA of each bacterium was extracted using an EZ1 automate (BioRobot) and the EZ1 DNA tissue kit (Cat No./ID: 953034, Qiagen, Hilden, Germany) according to the manufacturing protocol. The genomic materials were quantified using a Qubit assay (Life Technologies, Carlsbad, CA, USA) and then amplified by standard PCR. The standard PCR protocol was performed in a Thermal Cycler Peltier PTC200 cycler thermal model (MJ Research Inc., Watertown, MA, USA). Each reaction was conducted in a final volume of 50 μL, containing 5 μL of DNA from each sample, 25 HotstarTaq—AmpliTaq Gold (Life Technologies, Carlsbad, CA, USA), 1.5 μL of primers (Fd1-AGAGTTTGATCCTGGCTCAG; Rp2-ACGGCTACCTTGTTACGACTT (Eurogentec, Angers, France))^78^ and 17 μL DNAse/RNAse-free water. The amplification was performed as follows: an initial denaturation step at 95 °C for 15 min, 40 cycles of denaturation at 95 °C for 30 s, step hybridization at a temperature of 52 °C for 30 s, and elongation at 72 °C for 60 s. All PCR products were resolved in 0.5× Tris Borate EDTA buffer (Ref. ET020-A, EUROMEDEX, Souffelweyersheim, France) and 1.5% agarose (Ref. LE-8200-B, EUROMEDEX, Souffelweyersheim, France), purified using NucleoFast 96 PCR plates (Macherey–Nagel EURL, Hoerdt, France), and sequenced using the Big Dye Terminator Cycle sequencing kit (Perkin Elmer Applied Biosystems, Foster City, CA, USA) with an ABI Prism 3130xl Genetic Analyser capillary sequencer (Applied Biosystems, Bedford, MA, USA). The following primers were used for the sequencing of the complete 16S rRNA: Fd1-AGAGTTTGATCCTGGCTCAG; Rp2-ACGGCTACCTTGTTACGACTT; F536-CAGCAGCCGCGGTAATAC; R536-GTATTACCGCGGCTGCTG; F800-ATTAGATACCCTGGTAG; R800-CTACCAGGGTATCTAAT; F1050-TGTCGTCAGCTCGTG; and R1050-CACGAGCTGACGACA (Eurogentec, Angers, France). CodonCode Aligner software was used for alignment and assembly and to correct the sequence (https://www.codoncode.com/). A consensus sequence was generated after analysis. BLASTn searches were performed against the nr database to check the similarity of the sequence (https://blast.ncbi.nlm.nih.gov/Blast.cgi). A sequence similarity threshold of 98.65% by comparison with the phylogenetically closest species with standing in the literature was used to delineate species^79^.

## Supplementary Information

Supplementary Table S1.

Supplementary Table S2.

Supplementary Table S3.

Supplementary Table S4.

Supplementary Table S5.

Supplementary Table S6.
