# Supplementary material for: Culturomics revealed the bacterial constituents of the microbiota of a 10-year-old laboratory culture of planarian species S. mediterranea
Source: Sci Rep. 2021 Dec 21;11:24311. doi: 10.1038/s41598-021-03719-5 (PMC8692324; doi:10.1038/s41598-021-03719-5)
Supplement: Supplementary file 3 — Supplementary Table S3. [file 41598_2021_3719_MOESM3_ESM.docx]

**Table S3.** **Table S3.** Bacterial distribution of the laboratory strain [*S. mediterranea*](https://en.wikipedia.org/wiki/Schmidtea_mediterranea) starved for 2 weeks.

| **Phyla** | **Species** | **localisation** |
| --- | --- | --- |
| Actinobacteria | *Delftia acidovorans* | gut |
| Actinobacteria | *Pseudomonas fluorescens* | gut |
| Actinobacteria | *Herminiimonas contaminans* | gut |
| Actinobacteria | *Micrococcus luteus* | gut |
| Bacteroidetes | *Pseudomonas anguilliseptica* | mucus and gut |
| Bacteroidetes | *Pedobacter schmidteae* | gut |
| Bacteroidetes | *Agrobacterium tumefaciens* | gut |
| Bacteroidetes | *Micrococcus yunnanensis* | mucus and gut |
| Bacteroidetes | *Pedobacter ghigonii* | mucus and gut |
| Bacteroidetes | *Pseudomonas huaxiensis* | gut |
| Bacteroidetes | *Sphingomonas ginsenosidimutans* | gut |
| Firmicutes | *Pseudomonas gessardii* | gut |
| Firmicutes | *Pseudomonas sp.* Marseille-Q1929 | mucus and gut |
| Firmicutes | *Sphingomonas bisphenolicum* | mucus and gut |
| Firmicutes | *Sphingomonas paucimobilis* | gut |
| Proteobacteria | *Acinetobacter guillouiae* | gut |
| Proteobacteria | *Pedobacter wanjuense* | gut |
| Proteobacteria | *Pseudomonas brenneri* | mucus and gut |
| Proteobacteria | *Variovorax paradoxus* | mucus and gut |
| Proteobacteria | *Chryseobacterium balustinum* | gut |
| Proteobacteria | *Chryseobacterium scophthalmum* | mucus and gut |
| Proteobacteria | *Comamonas testosteroni* | gut |
| Proteobacteria | *Aeromonas hydrophila* | gut |
| Proteobacteria | *Aeromonas veronii* | mucus and gut |
| Proteobacteria | *Microbacterium oxydans* | gut |
| Proteobacteria | *Acinetobacter bereziniae* | gut |
| Proteobacteria | *Comamonas aquatilis* | gut |
| Proteobacteria | *Ensifer adhaerens* | gut |
| Proteobacteria | *Flavobacterium oncorhynchi* | gut |
| Proteobacteria | *Flavobacterium tructae* | mucus |
| Proteobacteria | *Rhizobium giardinii* | gut |
| Proteobacteria | *Shinella zoogloeoides* | gut |
| Proteobacteria | *Staphylococcus capitis* | gut |
| Proteobacteria | *Staphylococcus haemolyticus* | gut |
| Proteobacteria | *Acidovorax wautersii* | gut |
| Proteobacteria | *Metabacillus schmidteae* | gut |
| Proteobacteria | *Chryseobacterium schmidteae* | gut |
| Proteobacteria | *Corynebacterium lipophiloflavum* | gut |
| Proteobacteria | *Staphylococcus epidermidis* | gut |
| Proteobacteria | *Vogesella urethralis* | mucus |

Note: Epidermal mucus noted here mucus
