# Supplementary material for: Culturomics revealed the bacterial constituents of the microbiota of a 10-year-old laboratory culture of planarian species S. mediterranea
Source: Sci Rep. 2021 Dec 21;11:24311. doi: 10.1038/s41598-021-03719-5 (PMC8692324; doi:10.1038/s41598-021-03719-5)
Supplement: Supplementary file 5 — Supplementary Table S5. [file 41598_2021_3719_MOESM5_ESM.docx]

**Table S5.** Bacterial species detected in calf liver used to feed the laboratory strain of *S. mediterranea.*

| **bacterial species** | **phylum** |
| --- | --- |
| *Brochothrix thermosphacta* | Firmicutes |
| *Lactococcus piscium* | Firmicutes |
| *Pseudomonas frederiksbergensis* | Proteobacteria |
| *Pseudomonas gessardii* | Proteobacteria |
| *Serratia proteamaculans* | Proteobacteria |
| *Staphylococcus hominis* | Firmicutes |
| *Pseudomonas azotoformans* | Proteobacteria |
