# Supplementary material for: Culturomics revealed the bacterial constituents of the microbiota of a 10-year-old laboratory culture of planarian species S. mediterranea
Source: Sci Rep. 2021 Dec 21;11:24311. doi: 10.1038/s41598-021-03719-5 (PMC8692324; doi:10.1038/s41598-021-03719-5)
Supplement: Supplementary file 6 — Supplementary Table S6. [file 41598_2021_3719_MOESM6_ESM.docx]

**Table S6.** Bacterial species detected in water containing the laboratory strain *S. mediterranea.*

| **bacterial species** | **phylum** |
| --- | --- |
| *Aeromonas veronii* | Proteobacteria |
| *Chryseobacterium scophthalmum* | Proteobacteria |
| *Pseudomonas brennerii* | Proteobacteria |
| *Pseudomonas anguilliseptica* | Bacteroidetes |
